# Combined effect of body mass index and metabolic status on the risk of prevalent and incident chronic kidney disease: a systematic review and meta-analysis

**DOI:** 10.18632/oncotarget.10915

**Published:** 2016-08-10

**Authors:** Jian Zhang, Hong Jiang, Jianghua Chen

**Affiliations:** ^1^ Kidney Disease Center, The First Affiliated Hospital, College of Medicine, Zhejiang University, Hangzhou, P.R.China; ^2^ Kidney Disease Immunology Laboratory, The Third Grade Laboratory, State Administration of Traditional Chinese Medicine of PR China, P.R.China; ^3^ Key Laboratory of Multiple Organ Transplantation, Ministry of Health, Key Laboratory of Nephropathy, Zhejiang, P.R.China

**Keywords:** body mass index, obesity, metabolic status, chronic kidney disease, meta-analysis

## Abstract

**Objective:**

The risk of chronic kidney disease (CKD) differs in the normal weight, overweight, and obese individuals owing to metabolic abnormality. We aimed to determine the combined effects of body mass index (BMI) and metabolic status on the risk of the prevalence and incidence of CKD.

**Methods:**

Pubmed, Scopus, Web of science, and abstracts from recently relevant meetings prior to April 2016 were searched to identify eligible studies. Pooled relative risks (RR) and 95% confidence intervals (CI) were calculated using a random effects model.

**Results:**

Eight cross-sectional studies and four longitudinal follow-up studies with a total of 14787 and 166718 participants were separately included in present study. Compared with metabolically healthy normal weight individuals, metabolically healthy obese individuals showed increased risk for CKD, with RR of 1.235 (95%CI: 1.027 to 1.484), while metabolically health overweight individuals still presented in a healthy pattern, RR=1.094(95%CI: 0.774 to 1.547). In addition, metabolically abnormal groups had much higher risk for CKD, with RR of 1.572 (95%CI: 1.373 to 1.801), 1.652(95%CI: 1.139 to 2.397) and 1.898(95%CI: 1.505 to 2.395) across metabolically unhealthy normal weight, overweight and obese individuals respectively.

**Conclusion:**

Individuals with abnormal metabolic status are at a significantly elevated risk for CKD, regardless of BMI. For metabolically healthy individuals, CKD risk increases with the growth of BMI, and obese persons eventually have a higher risk.

## INTRODUCTION

Owing to its deadly adverse outcomes, including end-stage renal disease (ESRD) and increased cardiovascular risk, chronic kidney disease (CKD) has gradually became a major health burden worldwide [1–3]. As a consequence of lacking effective treatment for ESRD, devoting to identifying early risk factors of CKD is of great significance [4].

It has been well established that metabolic syndrome (MetS), a constellation of various metabolic abnormalities, is related to the development of CKD. Furthermore, the strength of association seems to increase along with the growth of the number of components of MetS [5].

Mounting evidence indicates that increased BMI is also associated with excess all-cause mortality and cardiovascular diseases [6]. Paradoxically, quite a few studies reported that obesity may confer a beneficial effect on individuals with chronic diseases [7–9]. Studies focused on the effect of obesity on the development of glomerulopathy generated discrepant results too [10–14]. Up to now, final conclusion has not yet been reached on such matters that if obesity itself without secondary metabolic abnormalities contributes to the development of CKD or whether the relationship between MetS and CKD differs along with weight change. The complexity of the relationship between BMI and CKD may presumably be attributed to the heterogeneity of obese phenotypes: presence or absence of concurrent metabolic abnormalities.

Combining obesity with different metabolic phenotypes generates the heterogeneity of obesity. Correspondingly, population can be divided into 6 sub-types: metabolically healthy with normal weight (MHNW), metabolically healthy overweight, metabolically healthy obese (MHO), metabolically abnormal with normal weight (MANW), metabolically abnormal overweight (MAOW), and metabolically abnormal obese (MAO) [15]. Among them, a clustering of risk factors including central adiposity, dyslipidemia, impaired fasting glucose, hypertension or insulin resistance (IR) has been defined as the MetS [16].

At present, existed studies yielded discrepant effects of obesity-metabolic subphenotypes on the risk of developing CKD. Additionally, data from these individual studies may be insufficient to demonstrate a possible differential risk for CKD.

So it is imperative for us to synthesize results of these related studies. Here, we first conducted a systematic review and meta-analysis of observational studies to determine the associations of BMI and the presence/absence of metabolic abnormalities with the risk of prevalent and incident CKD.

## RESULTS

**Figure 1:**
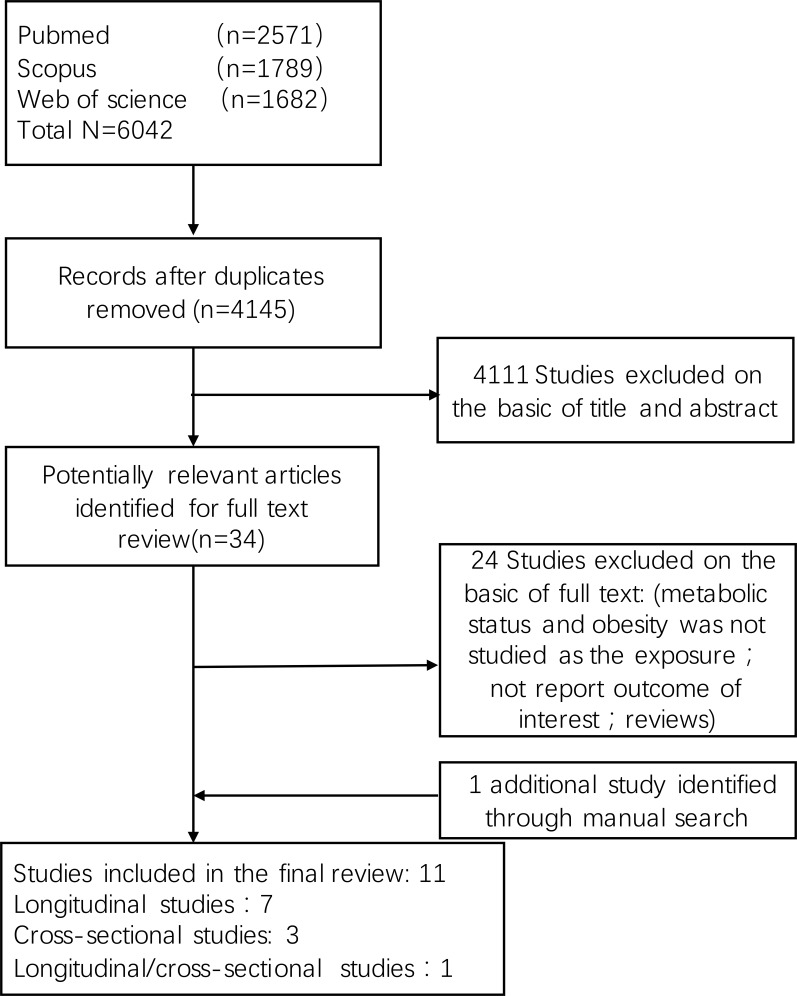
Flow diagram summarizing study identification and selection

Eleven studies met the inclusion criteria, of which three were cross-sectional studies [17–19], seven were longitudinal follow-up studies [20–26], and one study reported both follow-up data and prevalence of CKD [27]. A flow diagram of the study selection process was summarized in Figure 1. All studies were published in English. The total number of participants was 14787 and 166718 in cross-sectional and longitudinal studies respectively. Characteristics of included studies were shown in Table 1. For the present study, evaluating the follow-up outcome from eight longitudinal studies was the primary purpose. Seven of eight longitudinal studies chose incidence of CKD (eGFR < 60 ml/min per 1.73 m^2^) as the terminal event, while one study examined the joint associations of BMI and metabolic status with risk of ESRD (eGFR < 15ml/min per 1.73m^2^). Besides, three studies also reported the outcome of proteinuria separately. In the present review, we only pooled the outcome of development of eGFR < 60 or 15 ml/min per 1.73 m^2^. The multivariate adjusted RRs and 95% CI for the association between obesity-metabolic subphenotypes and incident CKD or ESRD risk was summarized.

**Table 1:** characteristic of cross-sectional studies and prospective cohort studies for the impact of obesity and metabolic abnormality on the prevalence and development of chronic kidney disease (eGFR < 60ml/min per 1.73 m2 and/or proteinuria)

Study, Year (reference)	Study type	Country	Study Name	Population	Participants No	Men, (%)	Mean Age ± SD or range (years)	Follow up (years)	Metabolic abnormality criteria	Measurement of obesity	Outcome	Definition of outcome
Chen et al., 2014 (26)	Cross-sectional study	China	-	Residents in the cities of Zhuhai and Guangzhou	2324	32.5%	52 ± 15 years	-	Insulin resistance NCEP ATPIII	BMI	Prevalence of CKD	CKD defined as eGFR < 60ml/min per 1.73m^2^ (MDRD) and/or urinary albumin to creatinine ratio (ACR)≥30 mg/g
Wang et al., 2014 (24)	Cross-sectional study	China	REACTION Study	Participants without CKD in Shandong Province	8586	31.5%	≥40 years old	-	Other	BMI; waist circumference	Mild reduced eGFR	eGFR between 30-59 ml/min/1.73 m^2^
Sesti et al., 2010 (27)	Cross-sectional study	Italy	CATAMERIS Study	Caucasians without kidney failure	440	41.8%	Aged 25-79 years old	-	Insulin resistance	BMI	Lower kidney function	eGFR between 30-59 ml/min/1.73 m^2^
Song et al., 2015 (34)	Cross-sectional/ prospective cohort study	Korea	Healthy Twin Study	Monozygotic and dizygotic twin individuals, and non-twin family members	3437/ 1881	40.8%/ 37.4%	43.9 ± 13.7; 44.1 ± 12.6 years	3.7±1.4 years	Modified NCEP ATPIII	BMI	CKD	eGFR <60 ml/min/1.73 m^2^(MDRD)
Mottaghi et al., 2015 (28)	Prospective cohort study	Iran	TLGS	Participants without CKD	5672	45.2%	40.4 ± 0.2 years	8.1 years	Modified NCEP ATPIII	BMI	Incident CKD	eGFR <60 ml/min/1.73 m^2^(MDRD)
Panwar et al., 2015 (25)	Prospective cohort study	United states	REGARDS	Participants without CKD	21840	45.2%	66.2 ± 10.2 years	6.3 years	Modified NCEP ATPIII	BMI	ESRD	eGFR<15/1.73 m^2^(CKD-EPI equation)
Junk et al., 2015 (29)	Prospective cohort study	korea	-	Participants without CKD	41194	60.5%	47.9(range, 20-87 years)	38.7 (range, 4.8-83.8 months)	Modified NCEP ATPIII	BMI	Incident CKD	eGFR < /1.73 m^2^(CKD-EPI equation)
Nishikawa et al., 2014 (30)	Follow up study	Japan	-	Participants without CKD	23894	85.9%	46.9 ± 10.2 years	7.8 years	NCEP ATPIII	BMI	Incident CKD	eGFR < 60ml/min/1.73 m^2^ or proteinuria was regarded as positive for dipstick urinary protein scores of ≥ 1+
Hashimoto et al., 2015 (31)	Follow up cohort study	Japan	Oike Health Survey	Participants without CKD	3136	58.5%	46.6 ± 9.2 years	8 years	IDF criteria	BMI	Incident CKD	GFR < 60ml/min/1.73 m^2^ or proteinuria, results for eGFR and proteinuria reported separately
Cao et al., 2015 (32)	Cohort study	China	-	Participants without CKD	6852	54.1%	47 (32-60) years	Average 54.3 months	Modified NCEP ATPIII	BMI	Incident CKD	eGFR <60ml/min /1.73 m^2^ or proteinuria
Chang et al., 2016 (33)	Cohort study	Korea	The Kangbuk Samsung Health Study	Metabolically healthy Participants without CKD	62249	50.5%	36.1 ± 6.6 years	369088 person-years	Insulin resistance modified NCEP ATPIII	BMI	Incident CKD	eGFR <60ml/min /1.73 m^2^(MDRD)

Abbreviations: CKD, chronic kidney disease; ESRD, end stage renal disease; BMI: body mass index; NCEP-ATP III, National Cholesterol Education Program's Adult Treatment Panel III; IDF, International Diabetes Federation; MDRD, Modified Diet in Renal Disease; CKD-EPI, Chronic Kidney Disease Epidemiology Collaboration equation.

Individual characteristics and prevalence of CKD were reported in four cross-sectional studies. Based on the definition of metabolically healthy and races of the included studies, distribution of MHO and MANW varied distinctly. Supplementary Table 1 shows the different distribution of participants in each group among studies.

### Obesity-metabolic subphenotypes and CKD risk

**Figure 2:**
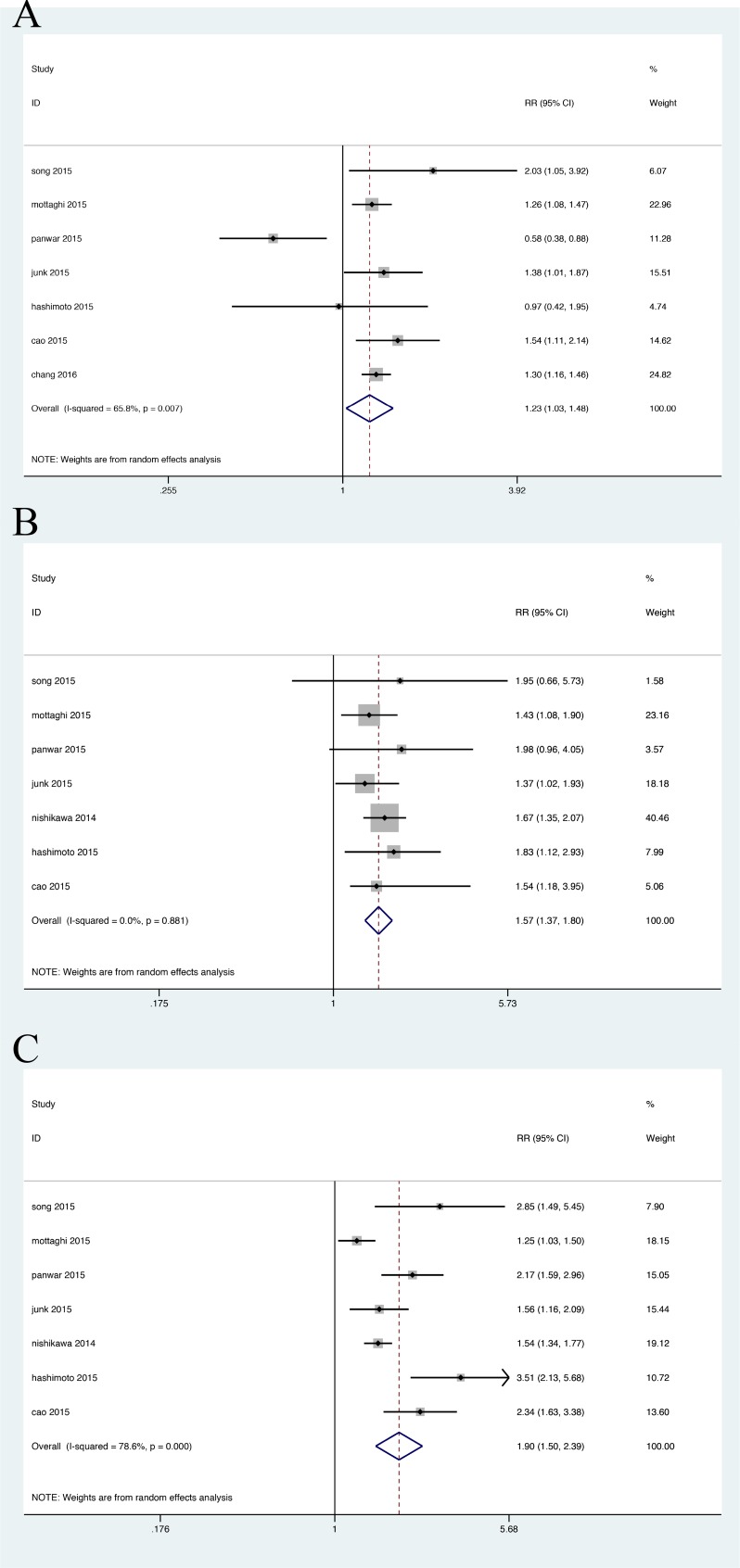
Relative risk of CKD in the obesity-metabolic subphenotypes **A**. Metabolic healthy obese group; **B**. Metabolic abnormal with normal weight group; **C**. Metabolic abnormal obese group.

A total of eight cohort studies were included for the analysis of incident CKD, of which one study reported the outcome of development of ESRD [21]. As Figure 2 shown, compared with metabolically healthy normal weight (MHNW), the pooled relative risks of CKD were extremely higher in metabolically unhealthy groups no matter companied with obese or not, with their RRs of 1.898(95%CI: 1.505 to 2.395) (Figure 2C) and 1.572 (95%CI: 1.373 to 1.801) (Figure 2B) separately. Furthermore, metabolically heathy obesity group were also at an increased risk estimate, with RR of 1.235 (95%CI: 1.027 to 1.484), results displayed in Figure 2A. To further elaborate such effect, we restricted to three studies that presented detail data about overweight group. Likewise, metabolically abnormal overweight group had comparably elevated risk of CKD, with RR 1.652(95%CI: 1.139 to 2.397) (Supplement Figure 1B); while in healthy overweight group, only a similar risk with MHNW group was observed) (Supplement Figure 1A).

Heterogeneity analysis was significant across the MHO group (*P* = 0.007, I^2^ = 65.8%); MHOW (*P* = 0.054, I^2^ = 65.7%); MAO (*P* = 0.000, I^2^ = 78.6%) and MAOW (*P* = 0.063, I^2^ = 63.8%) respectively. There was no heterogeneity in the MONW group (*P* = 0.881, I^2^ = 0%).

The sensitivity analysis of incident CKD after eliminating one study every time yielded similar effect sizes in magnitude and direction to the overall estimates. After the study by Panwar in which ESRD was regarded as the endpoint was excluded, remained risk estimate was calculated, and results revealed that the relative risks of incident CKD were not significantly changed, except the overweight group (Supplement Figure 2). Additionally, heterogeneity disappeared across the MHO group (*P* = 0.608, I^2^ = 0), but not in the MAO group (*P* = 0.000, I^2^ = 79.4%). Likewise, the risk estimates did not differ remarkably and high heterogeneity continued to exist by removing other studies one by one across the MAO group. Therefore, we attempted to perform subgroup analyses to further explore the heterogeneity in the MAO group, on the basis of definition on MetS, study duration, number and races of participants (Supplementary Table 2). However, significant heterogeneity remained.

Four cross-sectional studies reported the prevalence of CKD among MHNW, MHO, MANW and MAO populations at baseline. In the study of Wang, mildly reduced eGFR (60-90 ml/1.73 m^2^) was evaluated among different groups in middle-aged and elderly Chinese populations. Of which in the MHNW group, 19.21% of subjects had mildly reduced eGFR. In contrast, the corresponding proportions were 31.31% and 34.61% in MHO and MANW group. And there existed the highest increase in the proportion of mildly reduced eGFR (53.27 %) in the MAO group. Since we failed to obtain detail data of the study by Song after we tried to contact with authors, only the data from Chen and Sesti was pooled, with the overall prevalence of CKD in MHNW, MHO, MANW, MAO was 4.5%, 2.4%, 14.65% and 16.4% respectively. In summary, these findings were consistent with those from the above cohort studies in the general population.

### Study quality

There exists quite obvious difference of study quality among included studies. The study quality for each individual domain is presented in Table 2. Almost all studies were at low risk for bias for prognostic factor assessment and confounding. On the contrary, the included studies had different risk profiles for other domains such as study participation, study attrition, outcome assessment, and study analysis.

**Table 2:** Quality assessment of prospective cohort studies included in the systematic review

Study, year(reference)	Study Participation	Study Attrition	Prognostic Factor Measurement	Outcome Measurement	Confounding	Analysis	Variables that were Adjusted for
Mottaghi et al., 2015	Yes	Partly	Yes	Yes	Partly	Partly	basic Scr, age, sex, smoking, hypertension, and abdominal obesity
Panwar et al., 2015	Partly	No	Yes	Yes	Yes	Yes	age, race, sex, geographic region of residence, education, income, physical activity, current smoking, history of coronary heart disease, and history of stroke
Junk et al., 2015	Yes	Partly	Yes	Partly	Yes	Partly	age and sex, baseline glomerular filtration rate, history of cardiovascular disease, drinking, smoking, and exercise habits. alanine aminotransferase, γ-glutamyltransferase, low-density lipoprotein cholesterol, uric acid, and high-sensitivity C-reactive protein
Song et al., 2015	Partly	Partly	Yes	Partly	Yes	Partly	sex, alcohol use, smoking amount, and physical activity at baseline, household and sibling effects and sex
Nishikawa et al., 2015	Yes	Partly	Yes	Partly	Yes	Unclear	age, sex, smoking status, alcohol consumption, exercise habits, walking time in commutation, type of work and occupational exposure, C-reactive protein
Hashimoto et al., 2015	Partly	Partly	Yes	Partly	Partly	Yes	age, sex, smoking statues, alcohol use
Chang et al., 2016	Yes	Partly	Yes	Yes	Yes	Yes	age, sex, study center, and year of screening examination, smoking status, alcohol intake, and regular exercise
Cao et al., 2015	Yes	Partly	Yes	Yes	Yes	Yes	age, sex, smoking, plasma low-density lipoprotein cholesterol level, medication use, and physical inactivity

### Publication bias

No evidence of publication bias was detected in MHO, MHOW and MONW group on the basis of visual examination of the funnel plot or the Egger's test (*P* = 0.71, 0.486 and 0.462 respectively) (supplementary Figure S3). Possible publication bias was detected in reporting of studies for the MAOW, MAO group with incident CKD risk (*P* = 0.03 and 0.032 respectively).

## DISCUSSION

Recently, increasing interests have focused on the relationship between obesity-metabolic subphenotypes and various adverse events. Among them, there exist the biggest controversies over different outcomes in MHO group of obesity individuals who have relatively normal metabolic features and MANW group of metabolically unhealthy individuals whose BMI is in the normal level. Inconsistent effects of obesity-metabolic subphenotypes on the CKD risk were reported in various prospective longitudinal studies [28–31]. In addition, weight-loss intervention has also generated controversial results in some obesity-metabolic subtypes [32, 33].

So, the hypothesis that BMI combined with different metabolic states generate heterogenous CKD risk was addressed. Compared with MHNW persons, normal weight individuals with MetS and obese individuals at metabolically healthy status had significantly increased risk for incident CKD. As anticipated, obese people with MetS were at the highest risk of CKD. Only in the overweight group, different metabolic status led to discrepant risk for incident CKD. While remarkably elevated risk was found in MAOW individuals, it did not happen in overweight people with normal metabolic status.

Previous meta-analyses indicated that a higher BMI was associated with incrementally higher risk of kidney disease progress [34]. To some extent, these findings were in line with our results. But our analyses showed that overweight and even normal weight individuals may have higher risks for CKD if they stay in unhealthy metabolic status (RR = 1.652(1.139-2.397);1.572(1.373-1.801)), compared with MHO individuals(RR = 1.235(1.027-1.484)). These inconsistent results demonstrated the importance of metabolic status involved in the effect of obesity on the CKD risk.

In terms of question whether healthy pattern of increased weight is involved in the development of CKD, our present study indicated that only MHOW individuals had no obvious elevated risk for CKD. Existed evidence demonstrated excess visceral adipose tissue could result in a feedback loop where obesity induced declines in kidney function lead to a further development of hypertension by activating the sympathetic nervous and renin-angiotensin systems [35]. Computed tomography and magnetic resonance imaging results also suggested that MHO individuals presumably had relatively lower levels of visceral adiposity and liver fat content [36, 37]. Another possible mechanism is associated with impaired mitochondrial biogenesis in adipocytes which are important contributors to energy balance and metabolic hemostasis. Low mitochondrial number and activity in adipose tissue has been suggested as underlying factors in MetS. And recent studies showed mitochondrial dysfunction was involved in obese-metabolic related diseases, as manifested in down regulation of mitochondrial biogenesis, oxidative metabolic pathways and OXPHOS proteins [38].

Our meta-analysis also showed that normal weight was unable to reverse the increased risk resulted from unfavorable metabolic status. Non-obese MS is considered to be characterized by body composition and fat distribution abnormalities (elevation of the visceral fat to subcutaneous fat ratio) as well as decreased insulin sensitivity [39]. Therefore, further identifying visceral adipose distribution condition will be of greater value for people with lifestyle-related diseases.

In addition, higher prevalence of CKD was observed in metabolically unhealthy group, especially in MAO individuals. But the conclusion can not be drawn so imprudently owing to the small number of cross-sectional studies.

## STRENGTHS AND LIMITATION

Strengths of our present study include the large sample size which makes it possible to discriminate moderate differences among the BMI-metabolic subgroups and finally yields a convincing outcome. Both prospective cohort studies and cross-sectional studies were included and then analyzed separately in this meta-analysis, which gave a comprehensive understanding of the association between obese-metabolic subphenotypes and CKD risk.

Limitation of this study should not be ignored as well. Firstly, all of included studies used BMI to categorize normal weight, overweight and obesity. BMI might not be the best indicator for underlying adiposity [40]. So, it is indispensable for subsequent studies to evaluate the extent of visceral adiposity accumulation besides BMI to decrease the possibility of misclassifying and elevate study validity.

Secondly, observational studies involved in this meta-analysis may contain unknown confounding factors. The risk for bias varies from low to high for every individual domain. Just as other results from observational studies, associations do not imply causality certainly. However, the consistency of our results along with biologic plausibility highlights their correlation to clinical practice and, more significantly, can serve as elementary data for future interventional studies.

Thirdly, duration of exposure to the current BMI and metabolic factors, longitudinal changes in BMI and metabolic status during the follow up period was not illustrated in most studies. Nevertheless, the potential confounding is probably small considering the possibility of transition of individuals to higher weight categories is much larger than transition to lower weight categories.

Fourthly, lacking of uniform classification of BMI and standard definition of metabolic abnormality may be partly responsible for between-study heterogeneity. In the present study, 25 kg/m^2^ was used as cut-point to distinguish obesity from normal weight individuals, for the majority of study populations come from Asia. Therefore, this result may not be fit for populations from other races. This needs to be further examined in subsequently large cohort studies based on other populations. Modified NECP ATPIII criteria was most commonly used in included studies, including increased central adiposity, elevated triglycerides, low HDL-C, hypertension and high fasting glucose. Besides, insulin resistance was also independently regarded as a sign of metabolic abnormality in partial studies [19]. It was unable to conduct subgroup analysis for studies adopted IDF criteria and insulin resistance separately. Therefore, we restricted analysis only to those studies use modified ATPIII criteria, finding results were similar with that when all studies were pooled.

Limitations in statistical analyses also need to be considered. First, unadjusted estimates from the study of Chang were pooled in our meta-analysis. Besides, covariates such as physical activity, smoking status, possibly associated with CKD risk failed to be included for analysis. Second, heterogeneity was not fully explained in MAO group. Consequently, results from these estimates should be interpreted discreetly. At last, we could not fully exclude publication bias in MHO group, because we failed to get access to most unpublished results.

In conclusion, our present meta-analysis supports the opinion that the underlying heterogeneity for CKD risk exists in obese-metabolic subtypes. Except for MHOW individuals, obviously elevated risks for incident and prevalent CKD are observed in other obesity-metabolic groups. Moreover, all metabolically abnormal individuals are at a higher risk for CKD compared with corresponding healthy individuals. Thus, it is of great value to assess both BMI and metabolic status simultaneously for CKD risk estimate in high-risk populations. Relevant measures should also be made to prevent and control obesity and metabolic abnormality.

## MATERIALS AND METHODS

This systematic review and meta-analysis is conducted in accordance with the Meta-analysis of Observational Studies in Epidemiology (MOOSE) guidelines [41] and was registered at International Prospective Register of Systematic Reviews (number CRD42016038886).

### Eligible criteria

Studies were considered suitable for present meta-analysis if they met the requirement as below: conducted in adults; presented original cross-sectional or prospective data; evaluated participants according to categories of BMI, defined as normal weight (BMI < 25 kg/m^2^), overweight (BMI 25-29.9 kg/m^2^), and obesity (BMI ≥ 25 or 30 kg/m^2^); evaluated participants within these BMI categories according to different metabolic status (healthy or abnormal); and reported outcomes with prevalence and incidence of CKD, baseline characteristics, or all of above. Studies that were retrospective, did not stratify participants into the preceding 6 groups and lacked of valid data were excluded.

Due to the inconsistency across studies, we arbitrarily divided all participants into two groups primarily: defined normal BMI if it was identical or close to 18.5 to 24.9 kg/m^2^, and obesity if it was equal or close to ≥ 25kg/m^2^. Then we further distinguished overweight from obesity group, assigned their values between 25 to 29.9 kg/m^2^.

### Definition of metabolic syndrome

We included studies that used the following definitions of metabolic syndrome (MetS): modified NCEP-ATP III criteria, IDF criteria, presence of insulin resistance and other (Supplementary Table 3). According to the modified NCEP-ATP III criteria, metabolic syndrome was defined as having 3 or more of the following factors: (1)Elevated blood pressure: systolic/diastolic blood pressure ≥130/85 mmHg or ever received antihypertensive therapy; (2) Elevated triglyceride level: fasting triglyceride level ≥1.69 mmol/L (150 mg/dl); (3) Decreased HDL-C level: HDL-C < 1.04 mmol/L (40 mg/dl) in men or < 1.29 mmol/L (50 mg/dl) in women or ever received lipid-lowering medication; (4) Elevated glucose level: fasting glucose level ≥5.6 mmol/L (100 mg/dl) or ever received antidiabetic medication; (5) Obesity: waist circumference > 88 cm for women and > 102 cm for men. Insulin resistance was defined using the homeostasis model assessment (HOMA) [(fasting glucose × fasting insulin)/22.5]. A previously validated index (ISI) is derived from the OGTT [42].

### Data resources and search strategy

We identified all studies that reported the associations of obesity and different metabolic status with the risk of prevalent and incident CKD from 1950 to April 2016. Pubmed, Scopus, Web of science and abstracts from 2013 to 2015 meetings of Endocrine society and the European society of Endocrinology were searched using optimally sensitive search strategies. Additionally, manual review of reference lists of all included articles was also performed. The following combined text word and MeSH were used: (“metabolic subtypes” or “obesity subtypes” or “metabolic phenotype*” or “obesity phenotype*” or “metabolically healthy” or “metabolically unhealthy” or “metabolic abnorma*” or “metabolically obese” or obesity or “over weight” or “body mass index”) and (“chronic kidney diseases” or CKD or “End-Stage Kidney Disease” or ESRD), see Supplementary Search Strategy. All potentially eligible studies were retrieved and examined without the limit of languages. If the same cases were reported in more than one study, only the study with the most complete data was included.

### Data extraction and quality assessment

Two investigators carefully screened titles and abstracts from all eligible studies for relevance. Discrepancies between investigators were solved by a third reviewer until a consensus was achieved. Extracted data included the first name of authors, year of publication, country, study name, features of study population (sample size, age, proportion of men, baseline characteristics of participants), duration of the follow-up, classification of study groups, definition of metabolically unhealthy, ascertainment of outcomes, numbers of prevalent and incident CKD. Adjusted risk estimates (hazard ratio or relative risk [RR] or odds ratio [OR]) were extracted for the following mergence and further analysis.

All included studies were assessed for quality according to previously published guidelines [43]. Risk of biases mainly focus on the following six domains: (*1*) study participation, (*2*) study attrition, (*3*) prognostic factor measurement, (*4*) outcome measurement, (*5*) confounding measurement, and (*6*) statistical analysis. Each of these individual domains was graded as “Yes”, “Partly”, “No” or “Unclear”. The individual quality domain was categorized as low risk for bias (Yes) when complete information was reported to evaluate the study quality and the study reached the criteria for that quality domain, intermediate risk (Partly) was rated when the study reported inadequate data to assess that quality domain, and high risk (No) when it did not meet the criteria for that quality domain even if adequate data were reported. Studies without explicit description concerning the quality assessment were classified as “Unclear” and thus potentially at high risk for bias. Individual confounding factors adjusted in the multivariate analyses of the included studies were also covered in present study.

### Outcomes measures

The following outcome measures were suitable for present study and analyzed separately: (1) prevalence of CKD (eGFR < 60 ml/min per 1.73 m^2^) using the Modified Diet in Renal Disease equation or CKD-EPI equation, with exception in the study by Wang [18], eGFR between 60-90 ml/ min per 1.73 m^2^ was regarded as outcome. (2) development of eGFR < 60 ml/min per 1.73 m^2^ using the Modified Diet in Renal Disease equation or CKD-EPI equation, with exception in the study by Panwar [21], eGFR < 15 ml/min per 1.73 m^2^ was regarded as the event endpoint.

### Data synthesis and statistic analyses

Overall relative risk was calculated to assess the risk of incident CKD. Multivariable-adjusted risk estimates of dichotomous data were pooled and RRs were presented using the random effects model with inverse variance weighting because of heterogeneity between studies. In addition, a pooled incidence rate of cross-sectional studies was calculated for the prevalence of CKD at baseline.

I^2^ inspection was carried out to determine the magnitude of the variance among included studies. If the values of I^2^ were larger than 50%, moderate to extreme heterogeneity was judged. Next, subgroup analysis was conducted to explore the possible sources of variance among studies. Finally, we performed sensitivity by eliminating one study at a time and then calculated pooled relative risks for the residual studies to assess the degree to which the meta-analysis estimate depends on a particular study. The likelihood of publication bias was evaluated by using a funnel plot and the Egger's regression test. All tests were 2-sided and *P* value < 0.05 was considered statistically significant. All analyses were performed using STATA version 13.0 software (STATA Corp, College Station, Texas, USA).

## SUPPLEMENTARY MATERIALS FIGURES AND TABLES


