# Supplementary material for: Combined effect of body mass index and metabolic status on the risk of prevalent and incident chronic kidney disease: a systematic review and meta-analysis
Source: Oncotarget. 2016 Aug 10;8(22):35619–29. doi: 10.18632/oncotarget.10915 (PMC5482603; doi:10.18632/oncotarget.10915)
Supplement: Supplementary file 1 [file oncotarget-08-35619-s001.pdf]

Combined effect of body mass index and metabolic status on the risk of prevalent and incident chronic kidney disease: a systematic review and meta-analysis

Supplementary Material

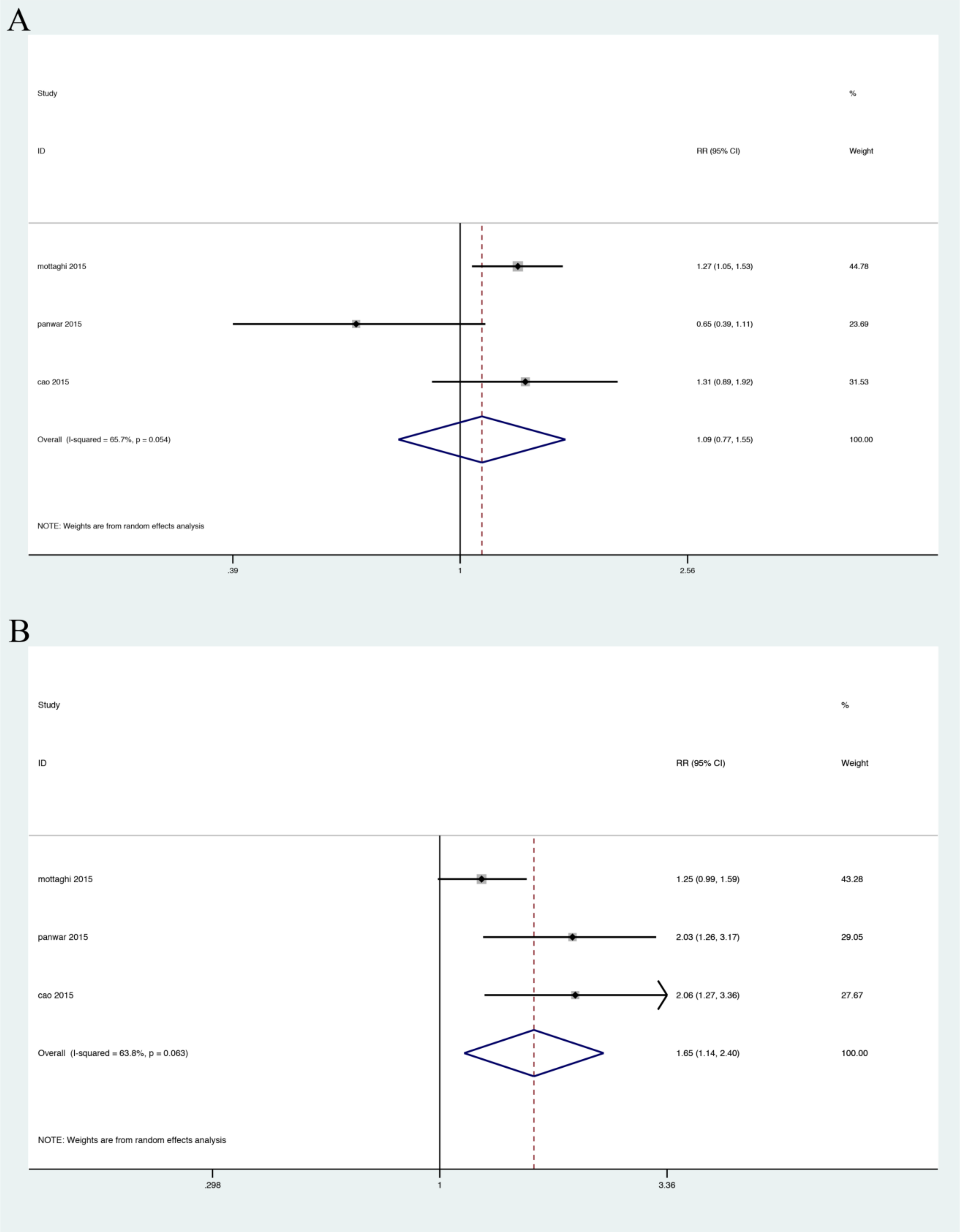

Figure S1. Pooled relative risk of CKD in metabolically healthy overweight (A) and metabolically abnormal overweight (B) individuals.

Metabolically healthy normal weight individuals were taken as the reference group.

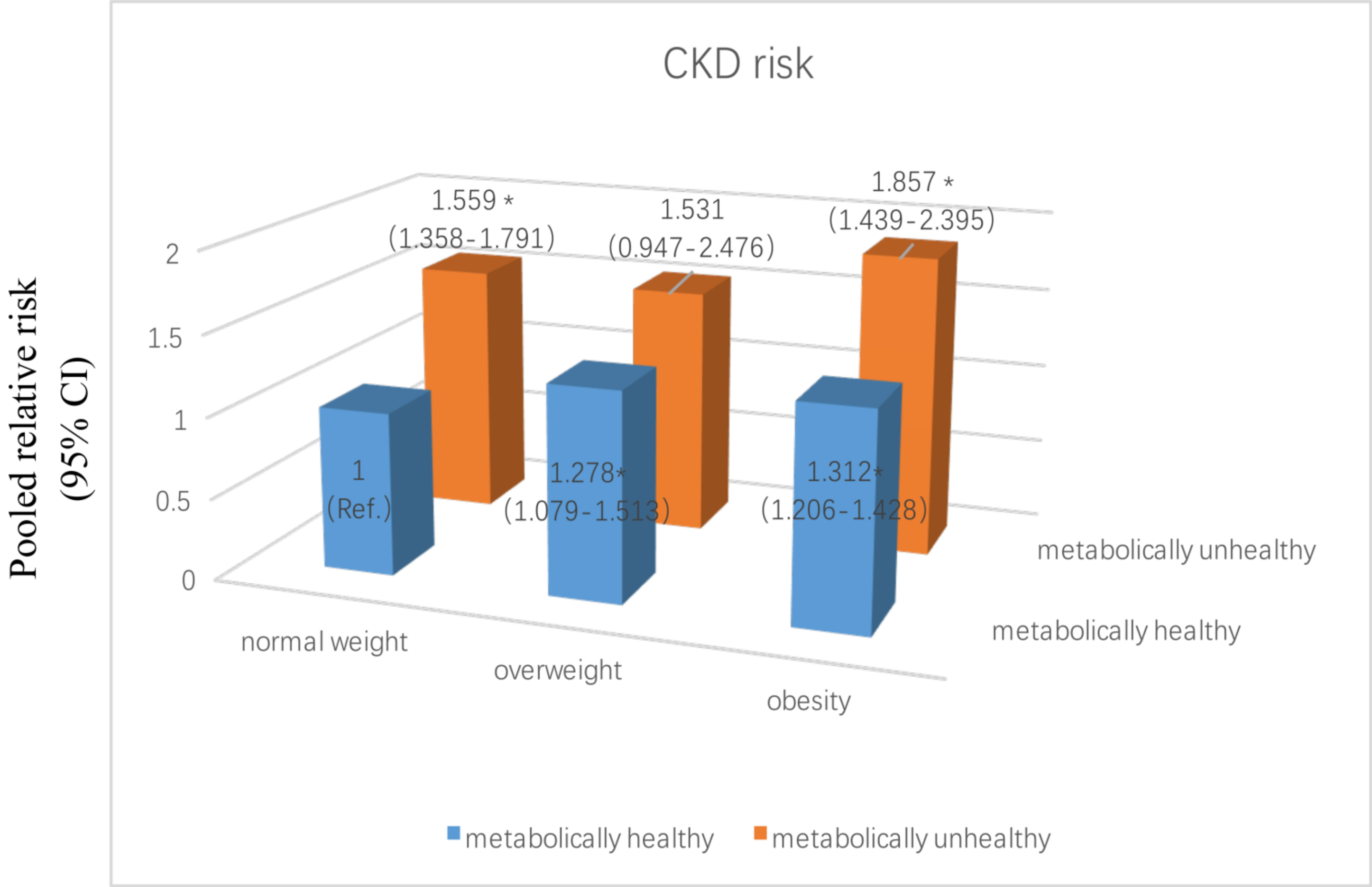

Figure S2. Analysis of pooled relative risk for CKD in obesity-metabolic subphenotypes after excluding the study from Panwar et al

The analyses were based on seven prospective studies, comprising 144878 participants and 3835 cases of incident CKD. *P < 0.05, pooled relative risk was significantly different from the reference group (metabolically healthy normal weight group).

Abbreviation: Ref, reference group; CI, confidence interval.

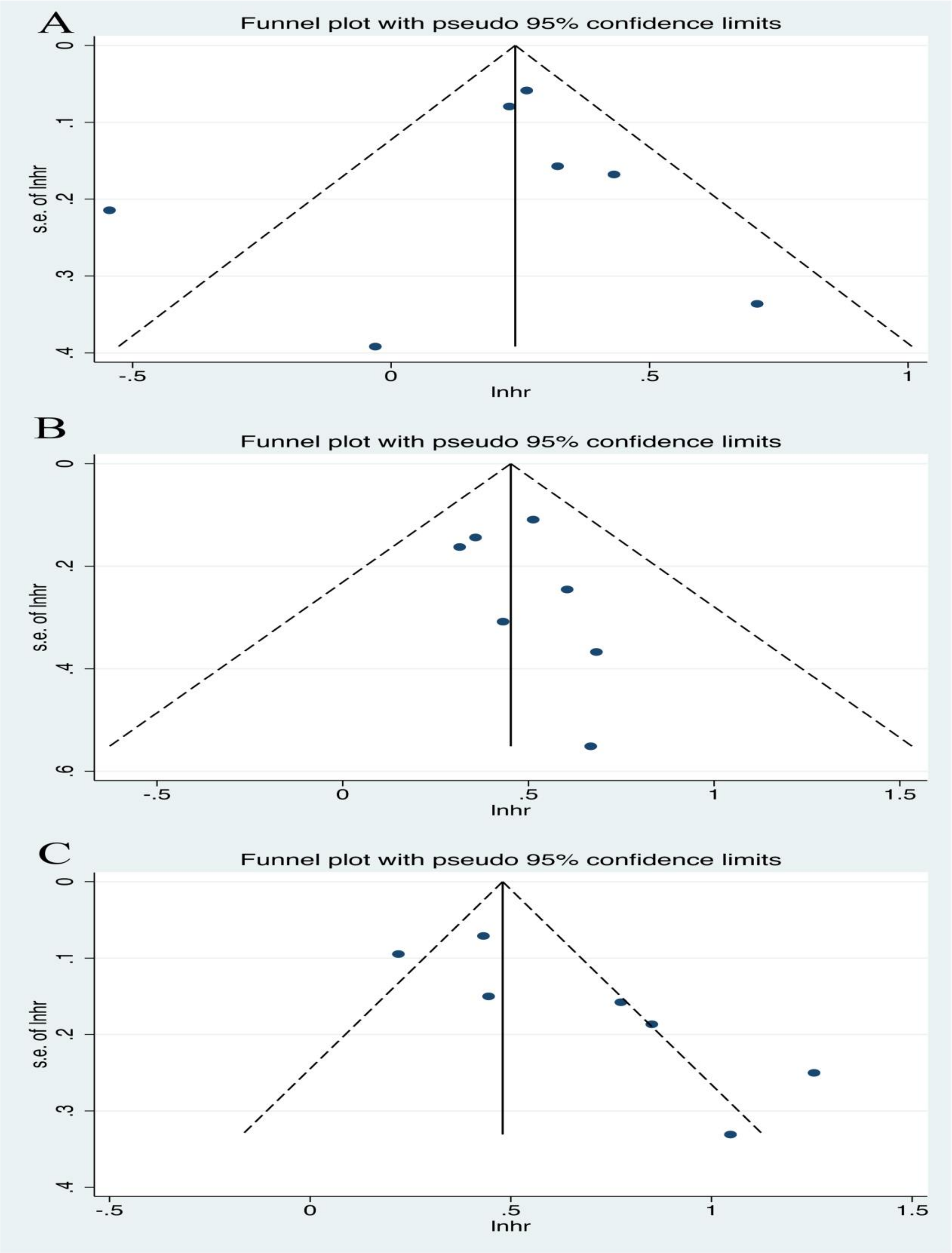

Figure S3. Funnel plot of relative risks of CKD in obesity-metabolic subphenotypes.

A: metabolically healthy obese individuals; B: metabolically abnormal normal weight individuals; C: metabolically abnormal obese individuals.

Supplementary table 1. Distribution of Participants, by Body Mass Index Category and Metabolic Status.

Study, Year （Reference）	Normal weight, n (%)		Overweight, n (%)		Obese, n（%）	
	Metabolically Healthy	Metabolically unhealthy	Metabolically Healthy	Metabolically unhealthy	Metabolically Healthy	Metabolically unhealthy
Chen et al, 2014(26)	432(18.6)	1017(43.8)	NA	NA	103(4.4)	772(33.2)
Wang et al. 2014(24)	713(8.3)	864(10.1)	NA	NA	1466(17.1)	5543(64.5)
Sesti et al, 2010(27)	122(27.7)		NA	NA	106(24.1)	212(48.2)
Song et al, 2015(34)	NA	NA	NA	NA	NA	NA
Mottaghi et al, 2015(28)	1817(32)	202(3.6)	1603(28.3)	786(13.9)	2103(37.1)	1550(27.3)
Panwar et al, 2015(25)	4688(21.5)	593(2.7)	5503(25.2)	2661(12.2)	8295(38)	8046(36.8)
Junk et al, 2015(29)	20329(49.3)	4835(11.7)	NA	NA	8587(20.8)	7443(18.1)
Nishikawa, et al,2014(30)	NA	650(2.7)	NA	NA	NA	2217(9.3)
Hshimoto, et al,2015(31)	2122(67.7)	445(14.2)	NA	NA	302(9.6)	267(8.5)
Cao et al, 2015(32)	3632(53)	232(3.4)	1852(27)	656(9.6)	2056(30)	932(13.6)
Chang et al,2016(33)	36490(58.6)	NA	13149(21.1)	NA	8149(13.1)	NA

NA: data not available.

Supplementary table 2. Risk for developing CKD among individuals with different obesity-metabolic subphenotypes based on various study characteristics.

subgroup	strata	Metabolically healthy obese group			Metabolically obese normal weight group			Metabolically abnormal obese group		
		n.	Relative risk (95%)	P-value	n.	Relative risk (95%)	P-value	n.	Relative risk (95%)	P-value
Definition of MetS	NECP-ATP III criteria	5	1.23 (0.91,1.66)	0.905	6	1.55 (1.35,1.79)	0.548	6	1.73 (1.41,2.13)	0.118
	Non-NECP-ATP III criteria	2	1.29 (1.15,1.45)		1	1.83 (1.12,2.93)		1	3.51 (2.13,5.68)	
Follow-up duration	＜ 5 years	3	1.50 (1.22,1.86)	0.169	3	1.44 (1.09,1.89)	0.483	3	2.04 (1.44,2.91)	0.704
	≥ 5 years	4	1.07 (0.83,1.39)		4	1.62 (1.39,1.90)		4	1.82 (1.33,2.49)	
Race	Asia	6	1.31(1.21,1.43)	0.014	6	1.62 (1.40,1.88)	0.392	6	1.99 (1.51,2.62)	0.551
	U.S.	1	0.58 (0.38,0.88)		1	1.37 (1.00,1.88)		1	1.56 (1.16,2.09)	
Number of participants	＜ 10000	4	1.34 (1.13,1.60)	0.373	4	1.54 (1.24,1.92)	0.832	4	2.24 (1.31,3.84)	0.464
	＞ 10000	3	1.06 (0.70,1.59)		3	1.59 (1.34,1.89)		3	1.90 (1.50,2.39)	

Supplementary table 3. Definitions of Metabolic syndrome (MetS)

Component	Modified NCEP-ATP III (25,28,29,30,32,34)	International Diabetes Federation (31)	Modified NCEP-ATP III plus insulin resistance （26,33）	Insulin resistance （27）	Other (24)
Hypertriglyceridemia	≥150 mg/dL	≥150 mg/dL	≥150 mg/dL	-	≥150 mg/dL
Low HDL cholesterol	< 40 mg/dL for men,<br>< 50 mg/dL for women	< 40 mg/dL for men,<br>< 50 mg/dL for women	< 40 mg/dL or men,<br>< 50 mg/dL for women	-	＜0.9 mmol/L (men),<br><br>＜1.0 mmol/L (women)
High blood pressure	≥130/85 mm Hg or documented use of antihypertensive therapy	≥130/85 mm Hg or treatment of previously diagnosed hypertension	≥130/85 mm Hg or documented use of antihypertensive therapy	-	≥140/90 mm Hg or documented use of antihypertensive therapy
High fasting glucose	≥110 mg/dL (note: modified in 2005 to be ≥100 mg/dL, or drug treatment)	≥100 mg/dL or previously diagnosed Type 2 diabetes	≥110 mg/dL (note: modified in 2005 to be ≥100 mg/dL, or drug treatment)	-	≥110 mg/dL (note: modified in 2005 to be ≥100 mg/dL, or drug treatment)
Abdominal/central obesity	Waist circumference: >102 cm in men, >88 cm in women	** Waist circumference: ethnicity specific values as below	-	-	-
High HOMA-IR score or low insulin sensitivity index （ISI）	-	-	> 2.69	ISI ≤ 61.3 mg $\times L^2 \times mmol^{-1} \times mU^{-1} \times min^{-1}$	-
**Criteria for metabolic abnormal**	**any 3 of the above 5 features**	**central obesity** plus 2 of the other components**	**At least one of above features**	**All of above features**	**At least one of above features**

HDL-C: High-density lipoprotein cholesterol; HOMA-IR: Homeostatic Model of Assessment-Insulin Resistance index assessment; ISI: insulin sensitivity index derived from OGTT.

** Ethnicity specific values for waist circumference in the International Diabetes Federation definition:

Ethnic group	***Men***	***Women***
Europids	>= 94 cm	>= 80 cm

South Asians	>= 90 cm	>= 80 cm
Chinese	>= 90 cm	>= 80 cm
Japanese	>= 90 cm	>= 80 cm
Ethnic south and central Americans	Use South Asian recommendations until more specific data available	
Sub-Saharan Africans	Use European data until more specific data available	
Eastern Mediterranean and Middle East (Arab) populations	Use European data until more specific data available	
